# Anterior knee schwannoma

**DOI:** 10.1093/jscr/rjz236

**Published:** 2019-09-07

**Authors:** Charmaine Ilagan, Lauren Poliakin, Armand Asarian, Philip Xiao, Sandeep Sirsi

**Affiliations:** 1 St. George’s University School of Medicine, True Blue, Grenada, WI; 2 Department of Surgery, The Brooklyn Hospital Center, Icahn School of Medicine at Mount Sinai, Brooklyn, NY 11201, USA; 3 Department of Pathology, The Brooklyn Hospital Center, Icahn School of Medicine at Mount Sinai, Brooklyn, NY 11201, USA; 4 Department of Surgery, New York University Langone Hospital Brooklyn, Brooklyn, NY 11220, USA

## Abstract

Peripheral nerve tumors are relatively uncommon with schwannomas being the most common type. Schwannomas are usually benign encapsulated tumors composed of neoplastic Schwann cells that generally do not transform to malignancy. Many are discovered incidentally as solitary tumors. The cause is unknown. Most occur spontaneously, while some develop in association with genetic disorders such as neurofibromatosis type 2 or schwannomatosis. Schwannomas can occur anywhere in the body. They affect all ages, with peaking incidence between ages 20 and 50 years, without predilection to sex or race. Many are asymptomatic; however, presenting signs and symptoms, such as paresthesia and pain, are due to mass effect and direct nerve invasion. Diagnosing includes combinations of thorough physical examination, imaging modalities such as magnetic resonance imaging and surgical biopsy. Treatment depends on factors such as location of the tumor and severity of symptoms. Asymptomatic patients are treated conservatively while symptomatic patients undergo surgical resection with favorable prognosis.

## INTRODUCTION

Schwannomas are the most common type of peripheral nerve tumors. They can arise from peripheral, spinal or cranial nerves. Though the cause is unknown, multiple schwannomas are known to develop from genetic disorders such as neurofibromatosis type 2, schwannomatosis or Carney complex. Commonly affected areas are the head and flexor surfaces of the upper and lower extremities and the trunk. Because of their slow growth, many patients are asymptomatic for months or years before experiencing symptoms. Typical presenting signs and symptoms of pain, paresthesia or weakness are due to direct nerve invasion, mass effect, involvement of surrounding tissues and disfigurement [1]. Therefore, neurological examinations assessing reflexes, motor and sensory functions are vital. Diagnosing can be difficult due to their slow growth and their mimicry of symptoms causing various diseases. Imaging tests such as computed tomography or X-rays are performed but magnetic resonance imaging (MRI) is the most beneficial imaging modality to aid in the diagnosis of schwannoma. T2-weighted MRI shows schwannomas as well as circumscribed ovoid masses with high intensity signal [2]. Surgical biopsy is the definitive diagnosis. Furthermore, schwannomas have a strong immunoreactivity for S-100 protein. Treatment depends on multiple factors such as the location of the tumor, severity of symptoms and whether it is benign or malignant. Conservative treatment is done for those that are asymptomatic while surgical resection is done to remove symptomatic tumors or those that grow quickly. Radiation can be combined with surgery for malignant schwannomas. Recurrence is unlikely after surgical resection with complete and rapid relief of symptoms.

## CASE PRESENTATION

### History and examination

This 53-year-old male presented to our office with complaints of an enlarging right knee mass causing intermittent, vague pain for the past couple months. He denied trauma to right knee, numbness, tingling and weakness. He reported vague knee pain with knee flexion and to palpation. Patient had a 2 × 2 cm firm, tender, mobile mass on right anterior knee. A neurological examination revealed intact motor and sensation. No muscle atrophy or knee effusion is present on physical examination.

### Operation

Patient received general anesthesia prior to incision. A 3-cm skin incision was made on the mass, which was deepened down through soft tissues. The patellar capsule was opened and an oblong mass identified. The mass was dissected free from the patellar capsule and suture ligated at the base of the stalk.

### Postoperative course

**Figure 1:**
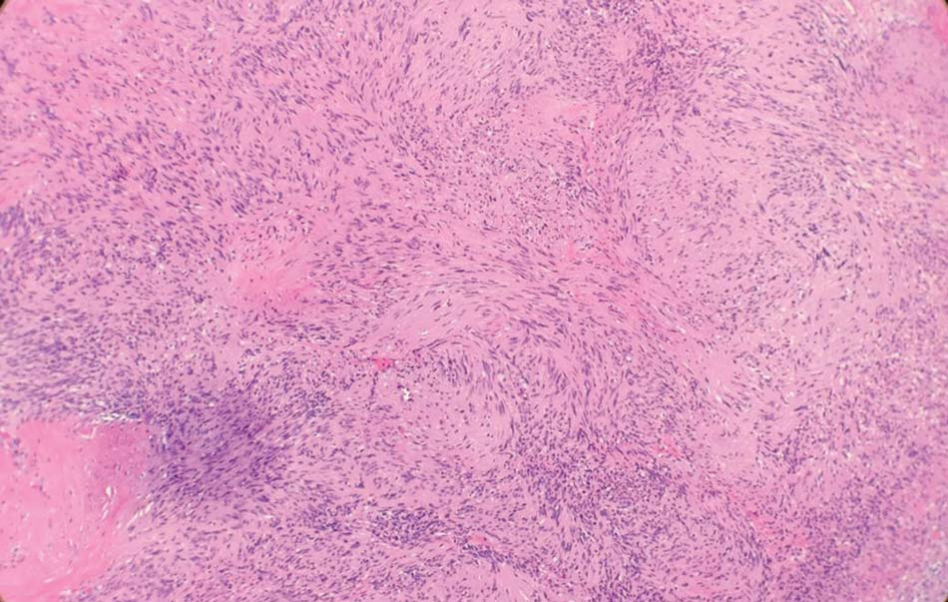
Microscopic examination reveals that tumor composed of biphasic spindle hypercellular Antoni A areas and hypocellular Antoni B areas (×2).

**Figure 2:**
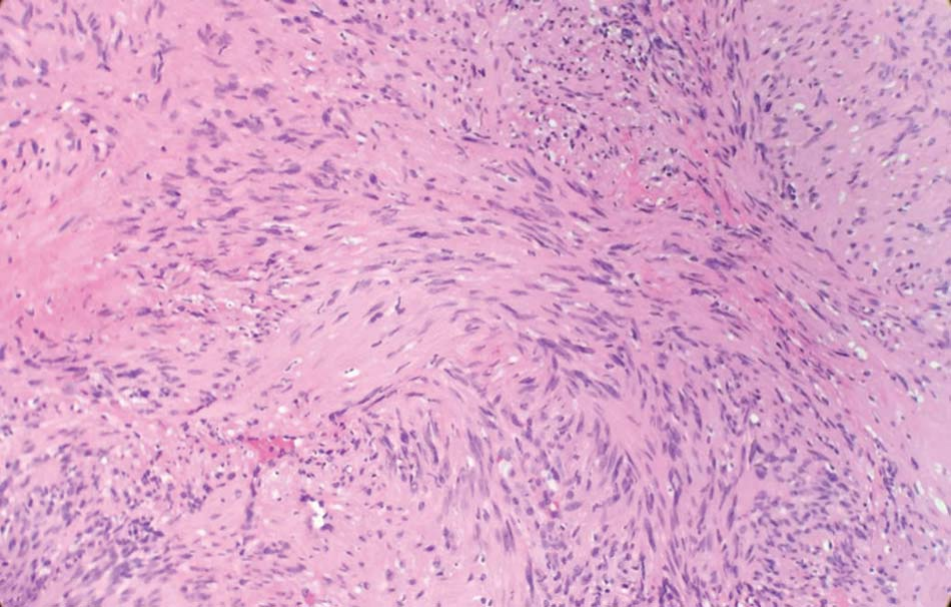
Higher magnification reveals spindle tumor cells are narrow, elongate, wavy with tapered ends interspersed with collagen fibers (×20).

**Figure 3:**
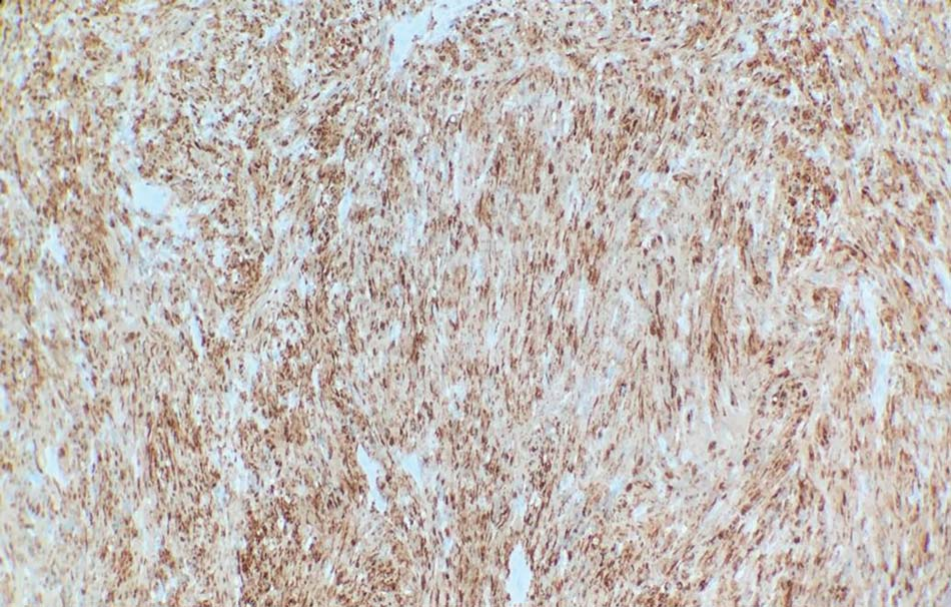
Immunohistochemical staining revealed that tumor cells are strongly positive for S-100 protein (×40).

Macroscopically, the tumor appeared encapsulated, rubbery and pink-tan in color. Microscopic examination reveals that tumor composed of biphasic spindle hypercellular Antoni A areas and hypocellular Antoni B areas (Fig. 1). Higher magnification reveals that spindle tumor cells are narrow, elongate, wavy with tapered ends interspersed with collagen fibers (Fig. 2). Immunohistochemical staining revealed that most tumor cells reacted strongly for S-100 protein (Fig. 3). Combined with immunohistochemical profile, these histological features are diagnostic of benign schwannoma. Patient was discharged home on the same day as the operation. At the 1-month follow-up, he was symptom free, without any pain or sensory disturbances.

## DISCUSSION

We report a case of an enlarging, anterior right knee mass associated with intermittent, vague pain. Differential diagnosis for unspecified anterior knee pain includes minor meniscal tear, Hoffa’s fat pad syndrome, quadriceps and patellar tendinopathy, Osgood–Schlatter disease, bursitis, Plica syndrome, lateral or medial collateral ligament sprain, patella subluxation, patellofemoral pain, chondromalacia patella and patellar stress fracture. Although the differential diagnosis of vague anterior knee pain is comprehensive, it can be limited with a detailed history, physical examination and appropriate use of MRI.

Schwannomas are rare, benign, often solitary, slow growing nerve sheath tumors. They are most often located in the brachial plexus (39%), followed by a slight predominance in the upper limits (30%) compared with their appearance in the lower extremities (24%) [3]. Most schwannomas are asymptomatic; it may become symptomatic after many months or years. Mass effect from the tumor can cause pain at the site of the lesion or radiating pain along the course of the involved nerve. On physical examination, one may notice a lump on the knee, and due to its vague symptoms, it makes it difficult to differentiate knee schwannomas from other soft tissue tumors. Ganglion cysts, lipomas and lipofibromatous hamartomas are usually easily compressible and soft to touch, whereas neoplastic tumors, like schwannomas, are firm to touch.

Diagnostic criteria for schwannoma requires a biphasic cellular pattern, consisting of two distinct regions namely Antoni A and Antoni B. Antoni A is hypercellular with eosinophilic cytoplasm, whereas Antoni B is hypocellular and has loose tissue comprising clear, vacuolated cytoplasm due to lipid accumulation. In addition, nuclear palisading is a typical feature and when pronounced forms Verocay bodies [4].

The occurrence of schwannoma is a rare finding, but the occurrence of anterior knee schwannoma is even more uncommon. There are multiple case reports of peroneal and tibial nerve schwannomas presenting as posterior knee masses. To date, there are few case reports involving anterior knee schwannoma. One previous case of anteromedial knee pain secondary to saphenous nerve schwannoma was initially misdiagnosed as meniscal tear [5]. Another documented case of anterior knee pain associated with a schwannoma of the saphenous nerve localized in the Hunter canal has been described [6].

## CONCLUSION

Anterior knee schwannoma is an extremely rare finding, only few cases have been noted. Currently, the cause is unknown, apart from incidences that develop from genetic disorders. Further research is required to understand its pathophysiology to have better diagnostic and treatment guidelines.

## Conflict of interest statement

None declared.
